# Local Delivery of Mesenchymal Stem Cells with Poly-Lactic-Co-Glycolic Acid Nano-Fiber Scaffold Suppress Arthritis in Rats

**DOI:** 10.1371/journal.pone.0114621

**Published:** 2014-12-04

**Authors:** Xiangmei Zhang, Kunihiro Yamaoka, Koshiro Sonomoto, Hiroaki Kaneko, Makoto Satake, Yuka Yamamoto, Masahiro Kondo, Jidong Zhao, Ippei Miyagawa, Kaoru Yamagata, Shunsuke Fukuyo, Yosuke Okada, Yoshiya Tanaka

**Affiliations:** 1 The First Department of Internal Medicine, University of Occupational and Environmental Health, Japan, Kitakyushu, Japan; 2 Integrative Technology Research Institute, Teijin Limited, Tokyo, Japan; 3 Pharmacology Research Laboratories I, Research Division, Mitsubishi Tanabe Pharma Corporation, Yokohama, Japan; University of Tokyo, Japan

## Abstract

Mesenchymal stem cells (MSC) have been used recently for the treatment of autoimmune diseases in murine animal models due to the immunoregulatory capacity. Current utilization of MSC requires cells in certain quantity with multiple courses of administration, leading to limitation in clinical usage. Here we efficiently treated collagen-induced arthritis rats with a single local implantation with reduced number of MSC (2∼20% of previous studies) with nano-fiber poly-lactic-co-glycolic acid (nano-fiber) scaffold. MSC seeded on nano-fiber scaffold suppressed arthritis and bone destruction due to inhibition of systemic inflammatory reaction and immune response by suppressing T cell proliferation and reducing anti- type II collagen antibody production. *In vivo* tracing of MSC demonstrated that these cells remained within the scaffold without migrating to other organs. Meanwhile, *in vitro* culture of MSC with nano-fiber scaffold significantly increased TGF-β1 production. These results indicate an efficient utilization of MSC with the scaffold for destructive joints in rheumatoid arthritis by a single and local inoculation. Thus, our data may serve as a new strategy for MSC-based therapy in inflammatory diseases and an alternative delivery method for bone destruction treatment.

## Introduction

Rheumatoid arthritis (RA) is an autoimmune disease with a worldwide incidence of approximately 0.5–1.0%, characterized by severe synovitis that results in articular destruction and affects activity of daily life [1]. Local immune response against collagen-rich joint components usually occurs in single articular, and eventually affects the majority of joints [2]. Once activated by inflammation, the synovial cells begin to form aggressive pannus which invade into cartilage and bone then develop the nonreciprocal damage [3]. Although the exact etiology of RA remains elusive, inflammatory cytokines, such as TNF-α, IL-6, IL-1β and IL-17, and autoreactive immune cells, including macrophages, T cells and B cells, play important roles in the pathogenesis [4]. Efforts to discover new target therapies have achieved considerable success such as the TNF-α inhibitors and B cell depleting therapies. However, current treatments do not provide joint repair and anti-inflammatory effect simultaneously in the synovium. Therefore, there is a necessity to develop a therapeutic strategy that could aim anti-inflammatory effect and subsequent joint repair.

Mesenchymal stem cells (MSC) possess multipotent capacity [5] and exhibit immunoregulatory properties [6]. In particular, MSC have inherently several advantages: they can be easily isolated from various organs, can differentiate into various types of cells, e.g., osteoblasts, chondrocytes and adipocytes, and generate regulatory T cells (Treg) which are guardian cells for maintaining immune tolerance. Meanwhile, accumulating evidences proved that the defective number or function of Treg play a crucial role during RA progression [7], [8]. In fact, the use of MSC has been reported to be safe and efficacious in a variety of autoimmune diseases, such as graft-versus-host disease (GvHD), systemic lupus erythematosus (SLE) and multiple sclerosis (MS) [9]–[11]. Therefore, the dual function of immune regulation and tissue repair prompted us to consider MSC as a new treatment tool for RA.

Until now, there have been conflicting reports of using MSC in treatment of rheumatic animal models. Multiple systemic administration of 1–5×10^6^ MSC/mice is essential to achieve therapeutic effect [12]. The results of RA patients treated with MSC also reported controversial results. One group reported intravenous (IV) injection of 1×10^6^ MSC/kg into 4 RA patients, while no one achieved the DAS-28-defined remission during the follow-up period [13]. The other report observed benefits by administration of 6–8×10^8^ cells through IV and/or intra-articular (IA) to 3 RA patients, but without long follow-up [14]. These previous treatments require large cell number, which is processed through numerous subcultures that could enhance the appearance of various cytogenetic abnormalities. Moreover, *in vitro* expansion longer than several weeks is reported to attenuate therapeutic effect [15] with decreased tissue repair ability [16]. However, MSC are the minority in their source tissues (bone marrow, adipose tissue or umbilical cord). Therefore, *in vitro* expansion is requisite and leads to the necessity for reducing cell number for MSC cell therapy.

The route of delivery is another key to consider MSC as a therapeutic tool. MSC originating from bone marrow lose their homing ability after a few hours *in vitro* culture [17]. Thus, even though one would hope the MSC to migrate to the target lesion after systemic injection such as IV or intraperitoneal (IP), it seems to be a difficult goal to achieve. Meanwhile, unlike other promising results in patients with GvHD or SLE, the relative unique and complex structure of the joints may be a challenge for MSC to migrate during arthritis. Thus, we considered direct delivery of MSC to the joint. However, previous studies reported the low efficacy of IA administration into arthritis model compared to IP [18], and the localization of MSC after infusion is poorly known [19]. Thus, to establish MSC as a realistic treatment tool for RA, we have developed a new delivery method that would force the MSC to reside at the implanted site maintaining their dual function.

For appropriate delivery of MSC into the inflamed lesion, we utilized the adhesive properties of MSC by using scaffold. In this regard, previous studies highlighted the advantages of poly-lactic-co-glycolic acid (PLGA) based on its controlled biodegradability and low immunogenicity [20], and applied PLGA as a carrier in drug delivery system [21] or as a scaffold for regeneration of bone defect [22]. Furthermore, the scaffold may enhance cell residence and cell differentiation [23]. We here developed a local delivery system of MSC by using nano-fiber PLGA (nano-fiber) as a scaffold.

## Materials and Methods

### Preparation of MSC

Bone marrow-derived human MSC (hMSC) (Lonza, Walkersville, US) were cultured with MSC growth medium (MGM) Bullet Kit (Lonza, Walkersville, US) in cell culture flasks at 37°C under 5% CO_2_ atmosphere. They were used in experiments after 75–85% confluence after 1–2 passages expanding within 3–7 days. Ethylene Diamine Tetraacetic Acid 0.01%/trypsin was utilized to release the cells from the culture flasks. Then hMSC were seeded directly on 24-well plastic plates or onto nano-fiber (Teijin, Tokyo, Japan) at a density of 2×10^4^ cells/cm^2^ or 2×10^5 ^cells/cm^2^ for later usage. Nano-fiber were produced into plain sheet with the 75–100 µm thickness then sterilized and packed in size of 1.0 or 2.0 cm^2^. Human skin fibroblasts (Lonza, Walkersville, US) were cultured with Dulbecco's Modified Eagle Medium (DMEM) supplemented with 10% bovine serum and 1% penicillin-streptomycin in the same condition with hMSC. Both hMSC and skin fibroblasts were purchased and originated from five different donors. Five donors for hMSC were 23–34 years old female and five donors for skin fibroblasts were 25–36 years old female.

### Treatment of collagen-induced arthritis (CIA) with human MSC through different delivery methods

To induce CIA, bovine type II collagen (CII) (Cosmo Bio, Tokyo, Japan) was emulsified in an equal amount of complete Freund’s adjuvant, and injected intradermally at 300 mL in the tail of 6–8 weeks old female Lewis rats (Charles River, Yokohama, Japan). They were provided with standard rat chow and water *ad libitum*. The clinical signs were monitored and the severity of arthritis, thickness of the hind paws and body weight were assessed by two investigators every 3 days. Arthritis severity was assessed in each limb using an established clinical score method [24] with a score of 0 to 4. Hind paw swelling was assessed by measuring the mean thickness of the ankles using 0.00–10.00 cm calipers. Body weight was checked using a balance with a precision of 0.01 gram. Nano-fiber were produced into plain sheet with the 75–100 µm thickness then sterilized and packed in size of 1.0 cm^2^. Rats with CIA were treated with hMSC inoculated through three different routes at the same time with immunization. 2×10^5^/cm^2^ hMSC were seeded onto 1.0 cm^2^ nano-fiber and incubated for 24 hours. Scaffold dimension 0.5 cm^2^ (0.5 cm×1.0 cm) combined with 1.0×10^5^ hMSC were implanted into ankles bilaterally (nano-hMSC) of 5 rats. The implantation was performed peri-articular, the whole scaffold resided outside the articular cavity and press-fitted to the articular capsule. For implantation, longitudinal incisions were performed through skin and muscles in dorsal ankles. The subcutaneous tissue was rare, the articular capsule was exposed directly after the skin was dissected. Setting the tibiotarsus articular in the center of the visual field, the hMSC with nano-fiber were inoculated to attach to the articular capsule. The other two delivery methods were intra-articularly (IA) and intra-peritoneally (IP) inoculation of hMSC. hMSC were suspended in phosphate-buffered saline (PBS) at 1×10^5^ cells/10 µl PBS. The IA group received 1×10^5^ hMSC per ankle to bilateral ankles (n = 5). IP group were inoculated with 2×10^5^ hMSC each rat (n = 5 rats). Within the 3 groups treated differently with hMSC (nano-hMSC, IA and IP), each animal was treated with 2×10^5^ cells total.

This study was carried out in strict accordance with the recommendations in the Guide for the Care and Use of Laboratory Animals of the University of Occupational and Environmental Health, Japan. The protocol was approved by the Committee on the ethics committee of the University of Occupational and Environmental Health (Permit Number: 08-014). All surgery was performed under sodium pentobarbital anesthesia and diethyl ether, and all efforts were made to minimize suffering.

### Evaluation of treatment outcome

Six weeks after immunization, the rats were sacrificed and evaluated by X-ray (Sofron, Tokyo, Japan) and micro-CT scanning (Hitachi Aloka Medical, Tokyo, Japan). The draining lymph nodes (LN) (inguinal and axillary LN) and spleen were collected at 2 or 6 weeks, their weight was measured then processed for histological examination. Tissue interleukin (IL) -1β, IL-6 and tumor necrosis factor (TNF)-α mRNA expression were analyzed by real-time polymerase chain reaction (PCR) around 2 weeks. Total RNA was purified using a RNeasy mini kit (Qiagen, Hilden, Germany) and total RNA (100 ng) was reverse-transcribed using the high capacity RNA-to-cDNA kit (Applied BioSystems, Foster City, CA) according to the specifications provided by the manufacturer.

Real-time PCR was performed in a StepOne Plus system (Applied BioSystems, Foster City, CA). Gene expression was analyzed with TaqMan Gene Expression Assay (Applied BioSystems, Foster City, CA) primer/probe pairs: GAPDH (Rn01775763_g1), IL-1β (Rn00580432_m1), IL-6 (Rn01410330_m1), TNF-α (Rn01525859_g1), IL-2 (Rn00587673_m1) and IL-17 (Rn01757168_m1), IFN-γ (Rn00594078_m1). The relative expression level of each gene was normalized to that of GAPDH, and analyzed using the 2(−Delta Delta C (T)) Method. Three times of biological replicates were used and each independent experiment was performed by triplicates.

IL-1β (rabbit polyclonal anti-rat IL-1β antibody, at 1∶50 dilution (ab9787, Abcam, Cambridge, UK)) expression in LN was evaluated by immunohistochemistry staining as previous report [25]. Horseradish peroxidase (HRP)-conjugated goat anti-rabbit secondary antibody (Nichirei, Tokyo, Japan) were used and the antigens were visualized using a 3,3-diaminobenzidine tetrahydrochloride (DAB) substrate (Dako, Carpinteria, US). Three times of biological replicates were used and each independent experiment was performed by triplicates. Serum anti-CII IgG were determined at 2 or 3 weeks by enzyme-linked immunosorbent assay (ELISA) (Chondrex, Washington, US). Three times of biological replicates were used and each independent experiment was performed by triplicates.

### Tracking implanted hMSC in CIA rat

The hMSC were transfected with green fluorescent protein (GFP)-carrying plasmid by electroporation using the human MSC nucleofector kit (Lonza, Walkersville, US). GFP-labeled hMSC were seeded on plastic plate or nano-fiber for 24 hours then used for treatment. Three days later, collected the tissues including ankle, spleen, LN, lung, liver and kidney. The hMSC were detected by GFP and human beta actin (ACTB) expression using PCR or anti-GFP immunohistochemistry staining (rabbit polyclonal anti-GFP antibody, at 1∶50 dilution (ab6556, Abcam, Cambridge, UK)). HRP-conjugated goat anti-rabbit secondary antibody was used and the antigens were visualized using a DAB substrate.

For PCR, the primer sequences and conditions were as follows: GFP, 5′- AGGACAGCGTGATCTTCACC- 3′ (forward) and 5′-CTTGAAGTGCATGTGGCTGT -3′ (reverse) (TM, melting temperature; 55°C, 35 cycles); human ACTB, 5′-AGCGAGCATCCCCCAAAGTT -3′ (forward) and 5′-GGGCACGAAGGCTCATCATT-3′ (reverse) (TM, melting temperature; 55°C, 35 cycles). The ladder for PCR electrophoresis is OneSTEP Marker 4 (Wako, Osaka, Japan). 2% agarose gel was used for electrophoresis. The suitable range of the ladder is 72 to1,353 bps. The size of GFP is 153 bps. Three times of biological replicates were used and each independent experiment was performed by triplicates.

### T cell proliferation and cytokine assay

CD4^+^ T cells were collected from the draining LN of each group around 2 weeks after treatment and purified using anti-rat CD4 beads (Miltenyi Biotec, Auburn, CA). 1×10^6^ cells were cultured in triplicate in total volume of 200 µl Roswell Park Memorial Institute (RPMI) 1640 medium supplemented with 1% normal rat serum and 1% penicillin-streptomycin in 96-well flat-bottomed plates with or without 5 µg/ml of phytohemaglutinin (PHA) (Sigma-Aldrich, St. louis, US) and incubated for 72 hours at 37°C. T cell proliferation was assessed by adding 0.5 µCi ^3^H-thymidine per well for additional 16 hours of incubation. Total RNA was isolated from CD4^+^ T cells after 24-hour culture, IL-2, IL-17 and interferon (IFN)-γ mRNA levels were analyzed by real-time PCR. The transcription factor Forkhead box P3 (Foxp3) positive cells were evaluated by immunohistochemical staining with anti- Foxp3 (Mouse monoclonal anti-FOXP3 antibody, at 1∶30 dilution (ab22510, Abcam, Cambridge, UK)) in ankles and inguinal LN harvested at 2 weeks. HRP-conjugated goat anti-mouse secondary antibody (Nichirei, Tokyo, Japan) were used and the antigens were visualized using a DAB substrate. Three times of biological replicates were used and each independent experiment was performed by triplicates.

### Measurements of transforming growth factor (TGF)-β1 production

MSC were seeded directly on 24-well plastic plates or onto 2.0 cm^2^ nano-fiber at a density of 2×10^4^ cells/cm^2^ and cultured in MGM. TGF-β1 mRNA levels were measured by real-time PCR after 24 hours culture. Gene expression was analyzed with TaqMan Gene Expression Assay (Applied BioSystems, Foster City, CA) primer/probe pairs: human TGF-β1 mRNA (Hs00998133_m1), human ACTB (Hs99999903_m1). The relative expression level of each gene was normalized to that of ACTB, and analyzed using the 2(−Delta Delta C (T)) Method. For detecting TGF-β1 protein, after 72 hours culture in MGM, the medium of MSC was replaced with serum-free MGM and cultured for another 24 hours, then the supernatant was collected and analyzed by ELISA (R&D, Minneapolis, US). Three times of biological replicates were used and each independent experiment was performed by triplicates.

### Statistical analysis

Data were expressed as mean±SEM. Differences between groups were analyzed by either the T test, or one-way ANOVA followed by post hoc Dunnett’s test. A *P* value<0.05 denoted the presence of a significant statistical difference. All statistical tests were conducted using The Statistical Package for Social Sciences (SPSS Inc., Chicago, US).

## Results

### Peri-articular implantation of MSC with nano-fiber improved CIA

**Figure 1:**
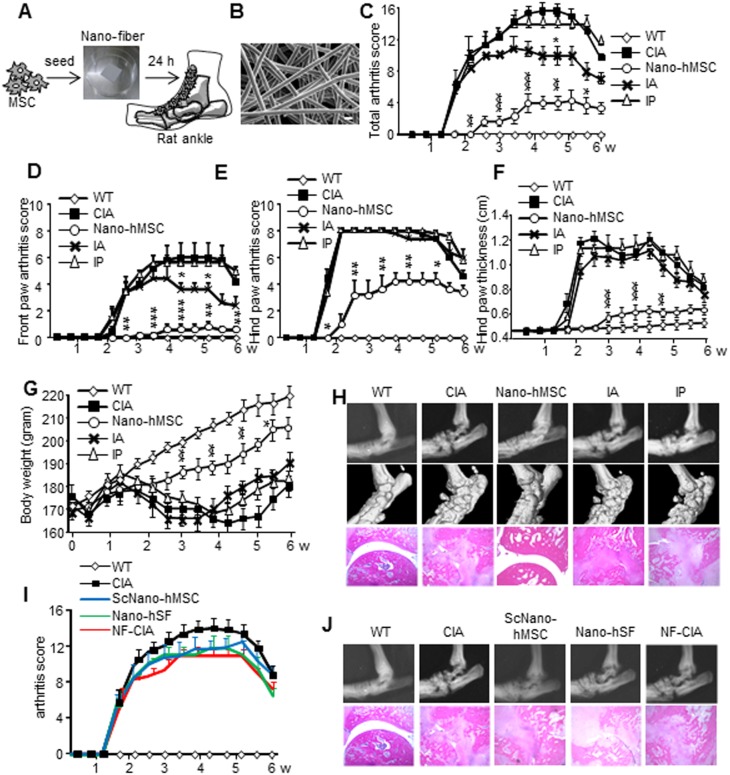
MSC in combination with nano-fiber suppresses arthritis and bone destruction. CIA were induced and treated by peri-articular inoculation of nano-fiber with human MSC (nano-hMSC) into ankles or injection of MSC intra-articularly (IA) or intra-peritoneally (IP) (2×10^5^ cells/rat) at the same time of immunization. (A): schematic diagram illustrated the method of treatment with nano-hMSC. (B): electron microscopic image of nano-fiber. Scale bar, 10 µm. Serial changes in (C): total arthritis score, (D): front paw arthritis score, (E): hind paw arthritis score, (F): hind paw thickness, and (G): body weight. (H): X-ray, micro-CT and hematoxylin and eosin (H&E) staining were performed at 6 weeks after immunization. (I, J): CIA rats were treated subcutaneous implantation of nano-hMSC into the dorsal region (ScNano-hMSC), peri-articular implantation of human skin fibroblasts in combination with nano-fiber (nano-hSF) or peri-articular inoculation of nano-fiber only (NF-CIA). Arthritis score, X-ray and H&E staining were performed. Data are mean±SEM of 5 rats in each group, *p<0.05, **p<0.01, ***p<0.001, versus CIA (by one-way ANOVA with post hoc Dunnett’s test). Three times of biological replicates were used and each independent experiment was performed by triplicates. Statistic analysis was performed every 6 days. Tests that showed no statistical difference are not marked (C–G, I). Arthritis severity was assessed in each limb with a score of 0 to 4. The score presented is the result of four paws in Figure 1C, two front paws in Figure 1D and two hind paws in Figure 1E. Representative pictures of 3 independent experiments were shown; original magnification ×40 (H, J). WT, wild type.

First, we assessed the clinical efficacy of different delivery methods of bone marrow-derived hMSC in rats with CIA. Schematic diagram of the method for peri-articular implantation of hMSC combined with nano-fiber PLGA scaffold, nano-hMSC treatment (Figure 1A) and the micro image of nano-fiber (Figure 1B) are shown. CIA developed at day 11 in CIA rats as well as rats treated with IA or IP of MSC. Serial examination demonstrated further exacerbation of CIA, even in the animals treated with IA or IP with a smaller effect in IA treatment. In contrast, nano-hMSC delayed the development of CIA from day 11 to day 15 and significantly suppressed the total arthritis score during the disease course compared to CIA and IA or IP (Figure 1C). An interesting finding is that local treatment of the ankles with nano-hMSC suppressed CIA not only in the hind paws but also in the front paws (Figure 1D, E). Measurements of hind paw thickness and body weight (Figure 1F, G) also reflected the limited effects of IA and IP, whereas nano-hMSC treatment resulted in significant decreases in both parameters, providing further support to the advantages of nano-hMSC treatment for CIA.

We have recently reported that hMSC can inhibit osteoclast differentiation by producing osteoprotegerin [26], and can also differentiate into osteoblasts in the presence of inflammatory milieu [27]. Therefore, we investigated the therapeutic effects of nano-hMSC on bone destruction. Radiographic examination showed severe destruction of the ankles in CIA at day 42, compared to the wild type (WT) control rats. Similar results were found in rats treated with IA or IP. In contrast, nano-hMSC almost completely suppressed bone destruction, showing images similar to those seen in WT rats (Figure 1H).

Histological analysis of the hind paw joint of CIA rats demonstrated the presence of inflammatory cells with synovial hyperplasia and pannus formation, together with severe destruction of the cartilage and bone. Similar results were noted in animals treated with IA or IP, whereas accumulation of inflammatory cells in rats treated with nano-hMSC was markedly reduced to the levels observed in WT (Figure 1H). Neither subcutaneous implantation of nano-hMSC into the dorsal region (ScNano-hMSC), peri-articular implantation of human skin fibroblasts in combination with nano-fiber (nano-hSF), nor implantation of nano-fiber alone (NF-CIA) had any effects on the CIA-related pathology (Figure 1I, J). The results demonstrated the clear benefits of nano-fiber used as a scaffold for administration of hMSC in CIA rats.

### Treatment of nano-hMSC suppressed systemic lymphoid tissue hypertrophy

**Figure 2:**
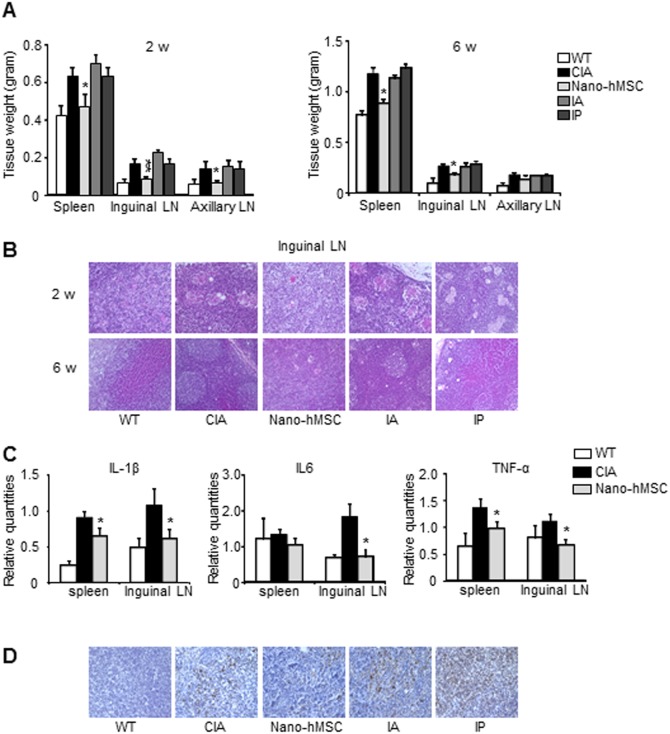
Nano-hMSC reduces systemic inflammation. CIA rats were treated as indicated. Lymphoid organs including spleen, inguinal and axillary LN were collected at 2 or 6 weeks after immunization. (A): Tissue weight was analyzed. (B): H&E staining of inguinal LN at 2 and 6 weeks were shown. (C): IL-1β, IL6 and TNF-α mRNA expression levels in the spleen and inguinal LN around 2 weeks were analyzed by real-time PCR. (D): IL-1β expression was assessed by immunohistochemistry staining in inguinal LN around 2 weeks. Values are mean±SEM of 5 rats in each group. *p<0.05, versus CIA (by one-way ANOVA with post hoc Dunnett’s test). Three times of biological replicates were used and each independent experiment was performed by triplicates. Tests that showed no statistical difference are not marked. Representative pictures from 3 independent experiments were shown, original magnification, ×200. WT, wild type.

Local treatment of the hind paws with nano-hMSC suppressed CIA not only in the hind paws but also in the front paws (Figure 1D, E). In support of this conclusion, the size and tissue weight of lymphoid organs were higher in CIA rats and rats treated with IA or IP, compared to WT rats. Furthermore, both the size and weight in rats treated with nano-hMSC were comparable with WT rats (Figure 2A). Histological examination of the lymph nodes (LN) at weeks 2 demonstrated the presence of multiple granuloma in CIA, IA and IP treated rats, suggesting the formation of germinal centers. In contrast, the findings in nano-hMSC-treated rats resembled those observed in WT rats, i.e., scarce lymphocyte accumulation and granuloma formation (Figure 2B). The reduced size of the draining LN and decreased number of germinal centers in the LN of nano-hMSC-treated rats was still observed by week 6, suggesting that the treatment was effective throughout the disease course. Moreover, such differences in histological findings were observed not only in the draining LN of the hind paws but also in the axillary LN, suggesting the systemic effects of nano-hMSC (figure S1). The mRNA levels of IL-1β, IL6 and TNF-α in the tissue obtained from both spleens and inguinal LN were decreased by implantation of nano-hMSC compared to those from CIA rats around week 2 (Figure 2C). Furthermore, tissue IL-1β expression, which plays an important role in CIA pathogenesis [24], increased in the inguinal LN harvested from CIA rats around week 2. A similar increase in IL-1β in LN was seen in IA- and IP-treated CIA rats, but implantation of nano-hMSC markedly reduced IL-1β expression (Figure 2D).

**Figure 3:**
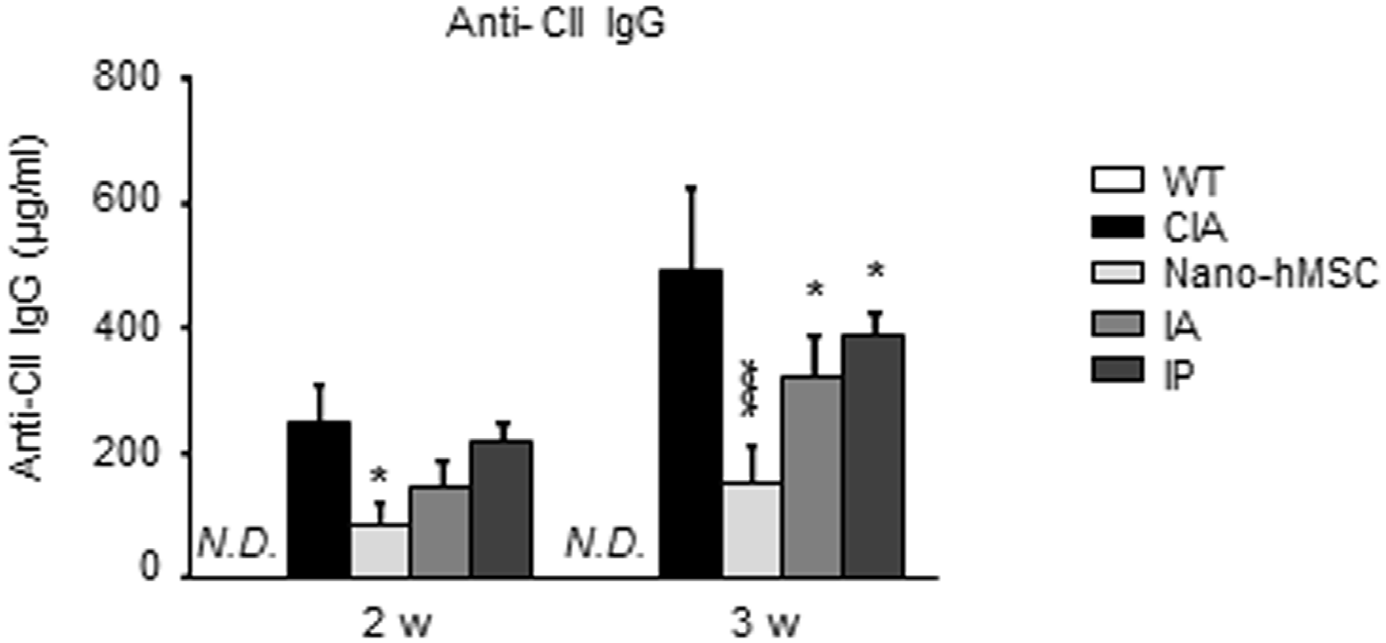
Local delivery of MSC with nano-fiber suppresses systemic immune response. CIA rats were treated as indicated. Serum samples of rats were collected on 2 and 3 weeks for measurement of anti-CII IgG concentration by ELISA. *p<0.05, **p<0.01, ***p<0.001, versus CIA (by one-way ANOVA with post hoc Dunnett’s test). Three times of biological replicates were used and each independent experiment was performed by triplicates. Tests that showed no statistical difference are not marked. N.D., not detectable; WT, wild type.

We next examined the serum levels of anti-CII IgG, representing the immunological response to CII. A significant decrease in anti-CII IgG was observed in CIA rats implanted with nano-hMSC at both week 2 and 3, whereas high-titer was observed at week 2 in IA- and IP-treated rats, comparable to CIA. However, anti-CII IgG decreased at week 3 in IA- and IP-treated CIA rats with less effect compared to nano-hMSC (Figure 3) presumably reflecting the necessity of multiple injection of high cell number to achieve treatment effect. These data suggested that nano-hMSC treatment suppress CIA and bone destruction through decreasing B cell activation and regulating both antigen-specific immunological reaction and inflammatory cytokine production.

### *In* *vivo* localization of hMSC post-implantation

**Figure 4:**
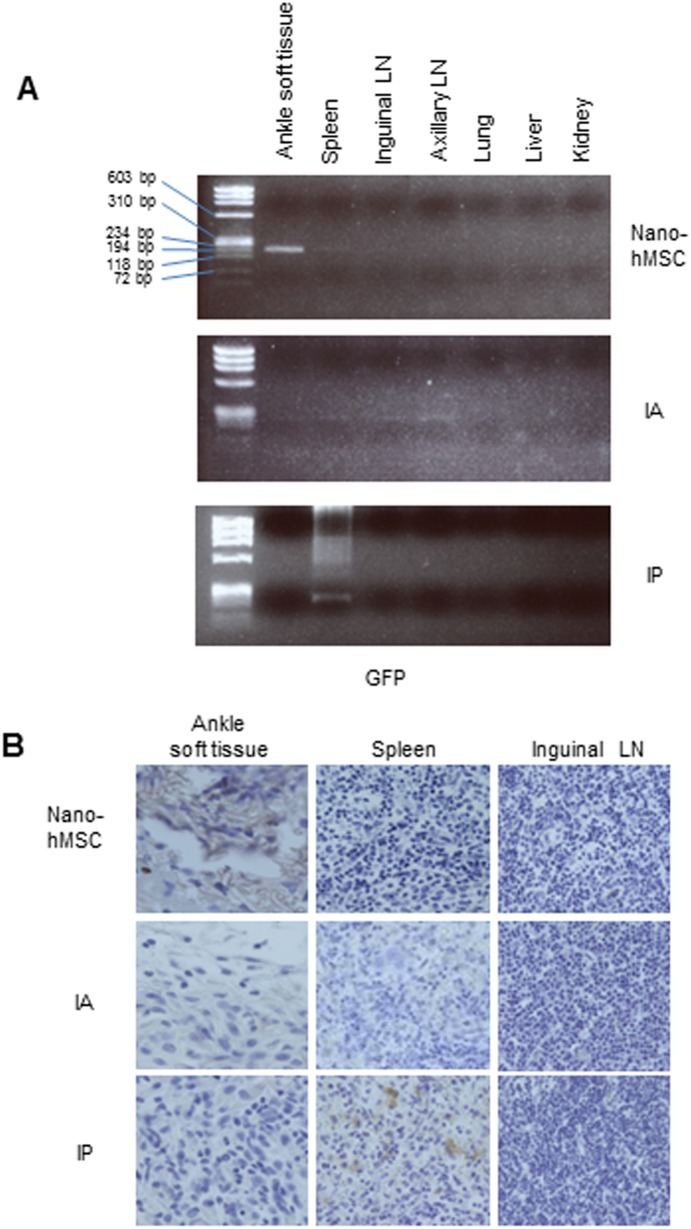
Inoculated MSC with nano-fiber resides at the site of implantation without systemic diffusion. MSC were transfected with a plasmid carrying GFP and seeded on nano-fiber or plastic plates and incubated for 24 hours. GFP+MSC were inoculated into bilateral ankles of CIA rats with nano-hMSC, IA or IP. Ankle, spleen, LN, lung, liver and kidney were collected 3 days after inoculation. GFP+ hMSC were detected by (A): PCR (the size of GFP is 153 bps) and (B): immunohistochemistry staining of GFP. Three times of biological replicates were used and each independent experiment was performed by triplicates. Representative pictures from 3 independent experiments were shown, original magnification×400. GFP, green fluorescent protein.

We next traced the location of hMSC *in vivo* in CIA rats treated with IA, IP or nano-hMSC, using hMSC transfected with plasmid encoding GFP. Ankle, spleen, LN, lung, liver and kidney were collected 3 days after hMSC inoculation. GFP and human ACTB mRNA were detectable only in ankle from nano-hMSC treatment and spleen from IP treatment (Figure 4A, figure S2). Immunohistochemistry staining with anti-GFP Ab revealed that GFP^+^ hMSC was only detectable in ankle of animals treated with nano-hMSC or spleen of animals treated with IP (Figure 4B). Therefore, nano-fiber presumably forced hMSC to reside at the implantation site.

### Nano-hMSC suppressed CD4^+^ T cell proliferation and cytokine production

**Figure 5:**
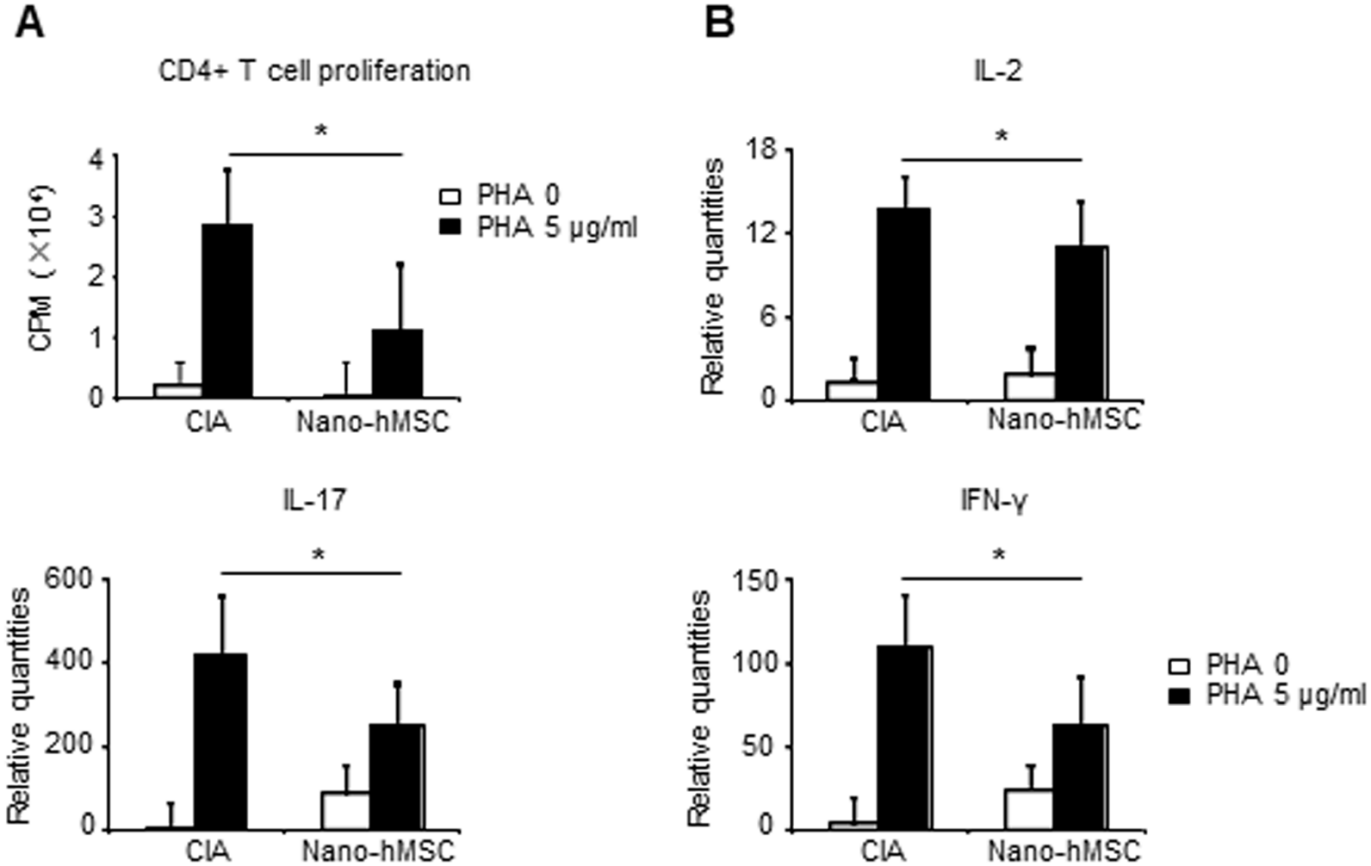
Nano-hMSC treatment suppresses CD4^+^ T cell proliferation and inflammatory cytokines expression. MSC in combination with nano-fiber were inoculated into bilateral ankles of CIA rats (nano-hMSC). Draining LN were collected around 2 weeks after immunization. (A): CD4^+^ T cells (1×10^6^/well) purified from LN by MACS were stimulated with phytohemagglutinin (PHA) for 72 hours (5 µg/ml). Cell proliferation was assessed by ^3^H thymidine uptake with an additional 16 hours. (B): IL-2, IL-17 and IFN-γ gene expression of CD4^+^ T cells was assessed by real-time PCR after stimulation with PHA (5 µg/ml) for 24 hours. Values are mean ± SEM of 3 independent experiments in each group. *p<0.05, versus CIA (by T test). Three times of biological replicates were used and each independent experiment was performed by triplicates. Tests that showed no statistical difference are not marked. CPM, counts per minute.

We assessed the effects of nano-hMSC on proliferation of CD4^+^ T cells isolated from the draining LN. CD4^+^ T cells obtained from CIA proliferated and expressed high level of cytokine mRNA, such as IL-2, IL-17 and IFN-γ in response to PHA (Figure 5A, B). However, T cell proliferation and cytokine expression were markedly suppressed in CD4^+^ T cells obtained from nano-hMSC treated rats. These results suggested, in addition to the direct anti-inflammatory effect of MSC, the involvement of regulatory CD4^+^ T cell subset that is induced by MSC. Therefore, we also analyzed the expression of Foxp3, a molecular marker characterizing immunoregulatory function of regulatory T cells (Treg) [28], [29] at week 2. Ankle images revealed the severe joint destruction with inflammatory cell infiltration with few Foxp3^+^ cells in CIA, IA or IP treatment group at week 2. Meanwhile, increased number of Foxp3^+^ cells was observed in ankles of nano-hMSC treated rats (figure S3A). Increased Foxp3^+^ cells were also observed in the inguinal LN from nano-hMSC treated rats compare to CIA at day 3, suggesting the systemic regulatory effect of nano-hMSC (figure S3B).

### Nano-fiber increased TGF-β1 production from MSC

**Figure 6:**
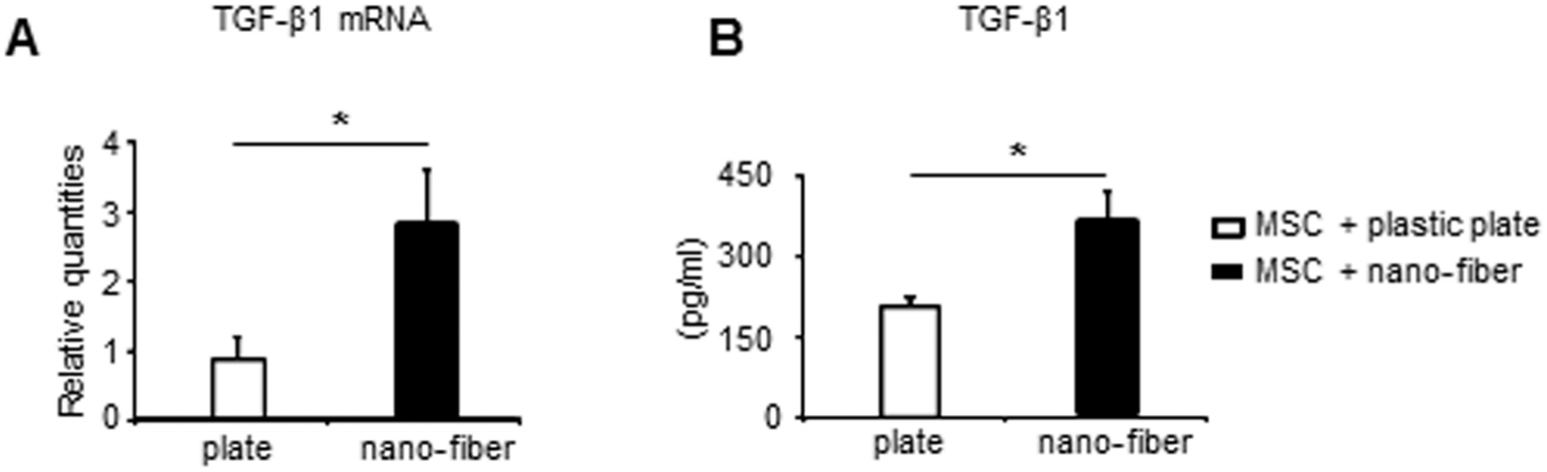
Nano-fiber increases TGF-β1 production from MSC. MSC were seeded onto plastic plates or nano-fiber scaffold and cultured in MGM. (A): TGF-β1 mRNA expression levels were analyzed by real time PCR after 24 hours culture. (B): After 72 hour-culture in MGM, the medium was replaced with serum-free MGM and incubated for another 24 hours, TGF-β1 concentration in the supernatant was measured. Values are mean±SEM of 3 independent experiments in each group. *p<0.05, **p<0.01, versus hMSC+plastic plate (by T test). Three times of biological replicates were used and each independent experiment was performed by triplicates.

TGF-β1 is a major immunomodulating cytokine secreted by MSC [6] and a crucial cytokine for differentiation of Foxp3^+^ Tregs [30]. Hence, we assessed the effects of nano-fiber on TGF-β1 production from hMSC. *In vitro* culture of hMSC on nano-fiber increased the expression level of TGF-β1 mRNA and TGF-β1 production, compared to those cultured on plastic plates for 24 hours (Figure 6A, B). These results indicate that the production of TGF-β1 was increased by MSC cultured on nano-fiber and efficiently induced Foxp3^+^ cells *in vivo*. Thus, MSC simultaneously suppressed the proliferation and cytokines production of CD4^+^ T cells.

## Discussion

We here observed that a single inoculation of a small number of MSC with nano-fiber scaffold into ankles of CIA rats significantly suppressed arthritis and bone destruction and the therapeutic effect was due to suppression of the systemic inflammatory reaction and immune response. Although MSC have been utilized for the treatment of autoimmune diseases in murine animal models, they are administered systemically with 1–5×10^6^ cells, leading to limitation in clinical usage. However, our *in vivo* tracing studies after inoculation of MSC transfected with GFP plasmid DNA showed that these cells remained within the scaffold and did not migrate to other organs, indicating successful treatment of rats with CIA by a single peri-articular inoculation of nano-hMSC.

*Ex vivo* experiments showed significant suppression of proliferation and cytokines production by T cells and in vitro cultured supernatant of MSC on scaffold contained high levels of TGF-β1, compared to MSC cultured alone. Previous reports also indicated that the major anti-inflammatory property of MSC is mediated by TGF-β1 [31], [32]. Daily intravenous or intraperitoneal injection of TGF-β1 has been shown to protect animals from the occurrence of CIA. However, administration of TGF-β1 was necessary prior to the onset of symptoms that was similar with our results requiring implantation at the time of immunization. Meanwhile, systemic TGF-β1 delivery resulted in unsatisfied result on suppression of sera CII IgG production indicating an alternative suppressive mechanism of nano-hMSC [33]. Furthermore, MSC are known to induce the development of Treg [34], [35], in which TGF-β1 plays an important role [36]. *In vitro* co-culture of MSC with CD4^+^ T cells isolated from the RA patients induces Treg [32] and *in vivo* administration of MSC also increases inducible Treg (iTreg) in the knee joints and draining LN [18]. These reports suggest the role of iTreg in the suppression of arthritic joints treated with nano-hMSC. Previous reports have indicated the quantitative and qualitative deficiency of Treg in RA [7], [8]. Interestingly, although adoptive transfer of CD4^+^CD25^+^ Treg cells was confirmed to be effective in CIA, anti-CII Ab and T cell proliferation were not affected [37]. In contrast, treatment with nano-hMSC reduced both anti-CII Ab and T cell proliferation, suggesting another though unknown mechanism for the anti-inflammatory effect in rats inoculated with MSC.

Although previous studies successfully inhibited CIA by MSC infusion, suppression of anti-CII IgG can only be observed in the late phase of CIA [18], [32], [38]. Our nano-hMSC treatment persistently suppressed anti-CII IgG production, further indicating the regulation of B cell functions. Since B cell responses are mainly T cell dependent, thus simultaneously supported the amelioration of T cells proliferation and cytokines production by nano-hMSC *ex vivo*.

It is also conceivable that the scaffold enhanced cell residence, providing a three-dimensional environment for MSC to survive, differentiate and take effects [39]. These benefits may make MSC inoculated with nano-fiber successfully decrease the arthritis. These characteristics of MSC inoculated with nano-fiber could have promoted the reduction in cell number observed in our experiments compared to previous reports (only 2–20%) [12], and explain the efficient and effective anti-inflammatory actions of nano-hMSC.

One of our interesting findings was that nano-hMSC implantation into the ankles prevented arthritis in the front paws. Although, front paw arthritis was prevented throughout the disease course, hMSC were detectable on nano-fiber only within the initial three days. In this regard, MSC survival after in vivo delivery is controversial [12]. Some reports have demonstrated that MSC would die or disappear after several days after *in* *vivo* administration, while others showed that MSC were detectable after 3–4 weeks in a murine arthritis models. In the former report, TGF-β1 secreted by local implanted hMSC may have contributed to suppression of arthritis in the front paws, however, we were not able to track hMSC after three days post implantation. Considering RA pathogenesis, locally accumulated synovial cells transform into active pro-inflammatory cells causing arthritis initially in local joints and later spreading to distant joints [4]. Likewise, CIA originally occurs in ankles then spread to front paw joints [24]. Thus, we speculate that TGF-β1 produced by hMSC residing at the locally implanted ankle for at least 3 days, suppressed the initial phase of CIA resulting in prevention of arthritis in the front paws.

One limitation of this study is that our treatment cannot be applied to human since therapy cannot be applied before disease onset. Actually, nano-hMSC implantation in developed CIA resulted in no obvious effects. This was presumably due to the systemically established immune response, such as increased anti-CII IgG antibody and pro-inflammatory cytokines, suggesting that local hMSC administration would require other supportive treatment. The cell number we used should also be taken into account since it was only 2–20% compared to previous reports. Interestingly, inoculation of MSC together with bortezomib after onset of CIA, the first proteasome inhibitor used clinically for the treatment of multiple myeloma, also resulted in efficient suppression of arthritis in RA animal models, and greatly enhanced the suppressive effects of MSC in the same model [40]. These features suggest that clinical use of nano-hMSC after the onset of the disease could still be feasible, since most RA patients demonstrate long-term alternation between exacerbation and remission.

Another limitation of our study is that we have utilized human MSC in CIA rats, representing xenogenic treatment. The efficacy of mice and human MSC in syngeneic, allogeneic and xenogenic usage in CIA mice was reported previously [12]. The ease and convenient usage of MSC is due to low immunogenicity including lower expression of MHC class II, which is beneficial for allogenic clinical usage in the future [6]. On the other hand, autogenic usage of MSC in RA patients can be considered. However, the functions and roles of MSC in the pathogenesis of RA are not clear at present. MSC from SLE patients are known for their low proliferation rate and IL-6 and IL-7 expression, suggesting that these two deficits could play important roles in the pathogenesis of SLE [41]. Therefore, treatment with allogeneic MSC from a healthy individual is presumably more doable treatment strategy.

Taken together, we demonstrated the beneficial effects of administration of nano-fiber PLGA scaffold as a delivery system of MSC into the arthritic joint. In this system, a single inoculation of a small number (2×10^5^) of MSC with nano-fiber resulted in long-lasting suppression of CIA rats. Both the *in vivo* and *in vitro* experiments suggest the importance of MSC residing at the local site of inflammation in the suppressing of inflammation and subsequent protecting of articular cartilage and bone. Our data developed a novel approach of MSC in cellular therapy for treatment of autoimmune and inflammatory diseases.

## Supporting Information

Figure S1 Nano-hMSC reduces systemic inflammation in axillary LN. CIA rats were treated as indicated. Axillary LN were collected at 2 or 6 weeks after immunization. H&E staining of inguinal LN and axillary LN were shown. Representative pictures from 3 independent experiments were shown, original magnification, ×200. WT, wild type.
(TIF)

Figure S2 Inoculated MSC with nano-fiber resides at the site of implantation without systemic diffusion. MSC were transfected with a plasmid carrying GFP and seeded on nano-fiber or plastic plates and incubated for 24 hours. GFP+MSC were inoculated into bilateral ankles of CIA rats with nano-hMSC, IA or IP. Ankle, spleen, LN, lung, liver and kidney were collected 3 days after inoculation. GFP+ hMSC were detected by PCR of human ACTB gene expression. Three times of biological replicates were used and each independent experiment was performed by triplicates. Representative pictures from 3 independent experiments were shown, GFP, green fluorescent protein.
(TIF)

Figure S3 Implantation of nano-fiber induce Foxp3+ cells. CIA rats were induced and treated as indicated. Foxp3+ cells were detected by immunohistochemistry staining in (A): ankles and (B): inguinal LN at 2 weeks. Representative pictures from 3 independent experiments, original magnification×400.
(TIF)
