# Supplementary figures and images for: Local Delivery of Mesenchymal Stem Cells with Poly-Lactic-Co-Glycolic Acid Nano-Fiber Scaffold Suppress Arthritis in Rats
Source: PLoS One. 2014 Dec 4;9(12):e114621. doi: 10.1371/journal.pone.0114621 (PMC4256456; doi:10.1371/journal.pone.0114621)

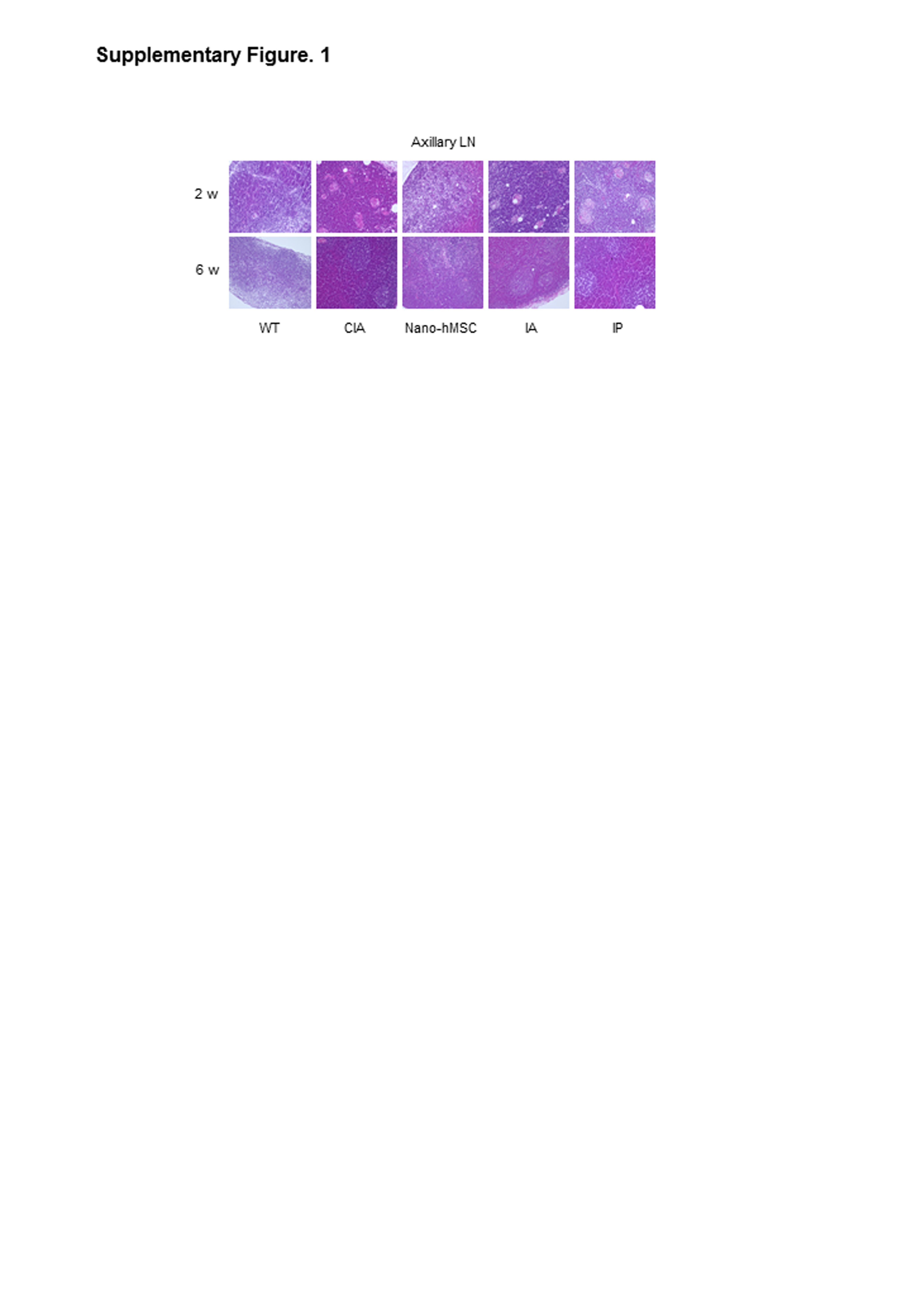

Supplement: Figure S1 — Nano-hMSC reduces systemic inflammation in axillary LN. CIA rats were treated as indicated. Axillary LN were collected at 2 or 6 weeks after immunization. H&E staining of inguinal LN and axillary LN were shown. Representative pictures from 3 independent experiments were shown, original magnification, ×200. WT, wild type. (TIF) [file pone.0114621.s001.tif]

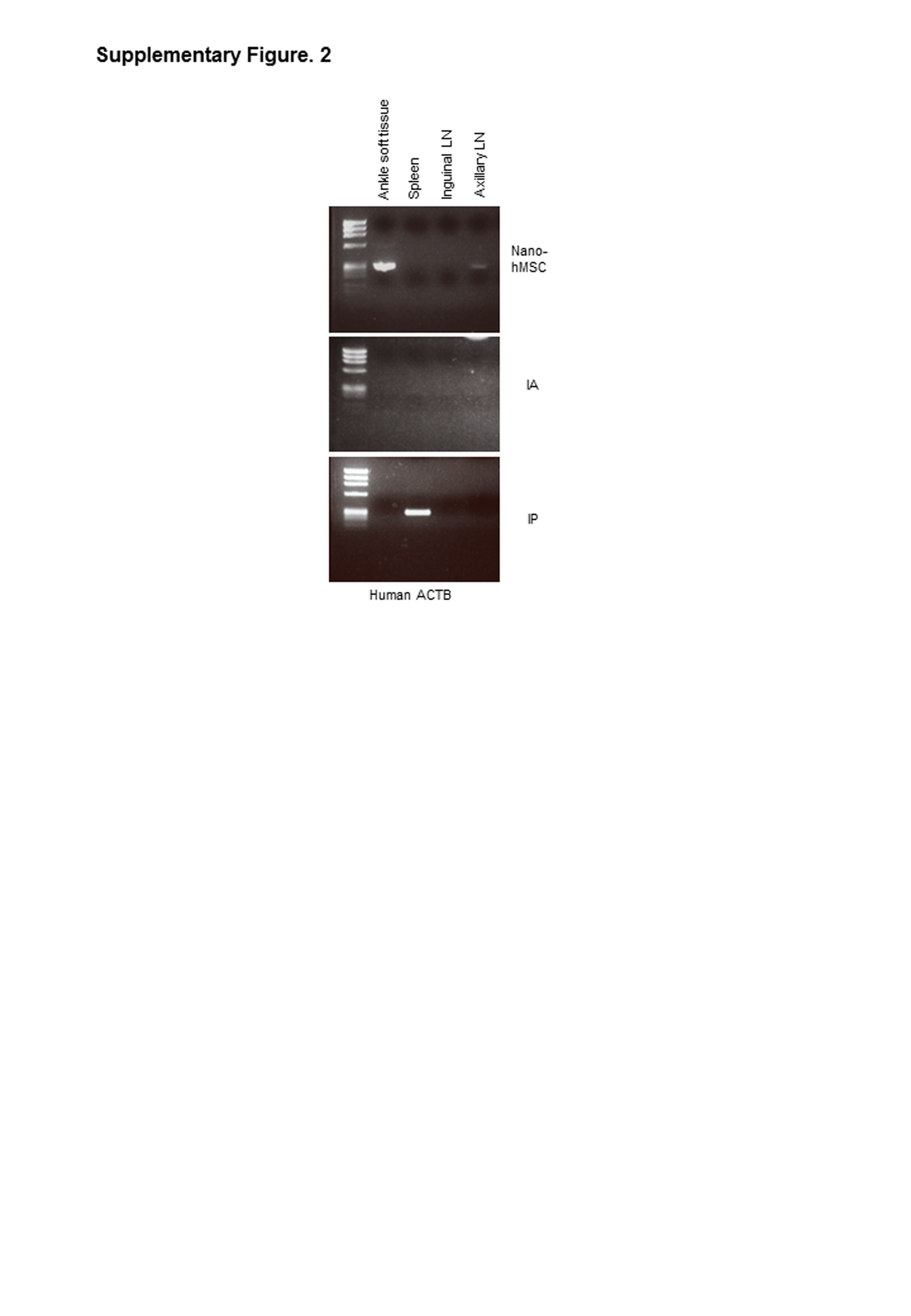

Supplement: Figure S2 — Inoculated MSC with nano-fiber resides at the site of implantation without systemic diffusion. MSC were transfected with a plasmid carrying GFP and seeded on nano-fiber or plastic plates and incubated for 24 hours. GFP+MSC were inoculated into bilateral ankles of CIA rats with nano-hMSC, IA or IP. Ankle, spleen, LN, lung, liver and kidney were collected 3 days after inoculation. GFP+ hMSC were detected by PCR of human ACTB gene expression. Three times of biological replicates were used and each independent experiment was performed by triplicates. Representative pictures from 3 independent experiments were shown, GFP, green fluorescent protein. (TIF) [file pone.0114621.s002.tif]

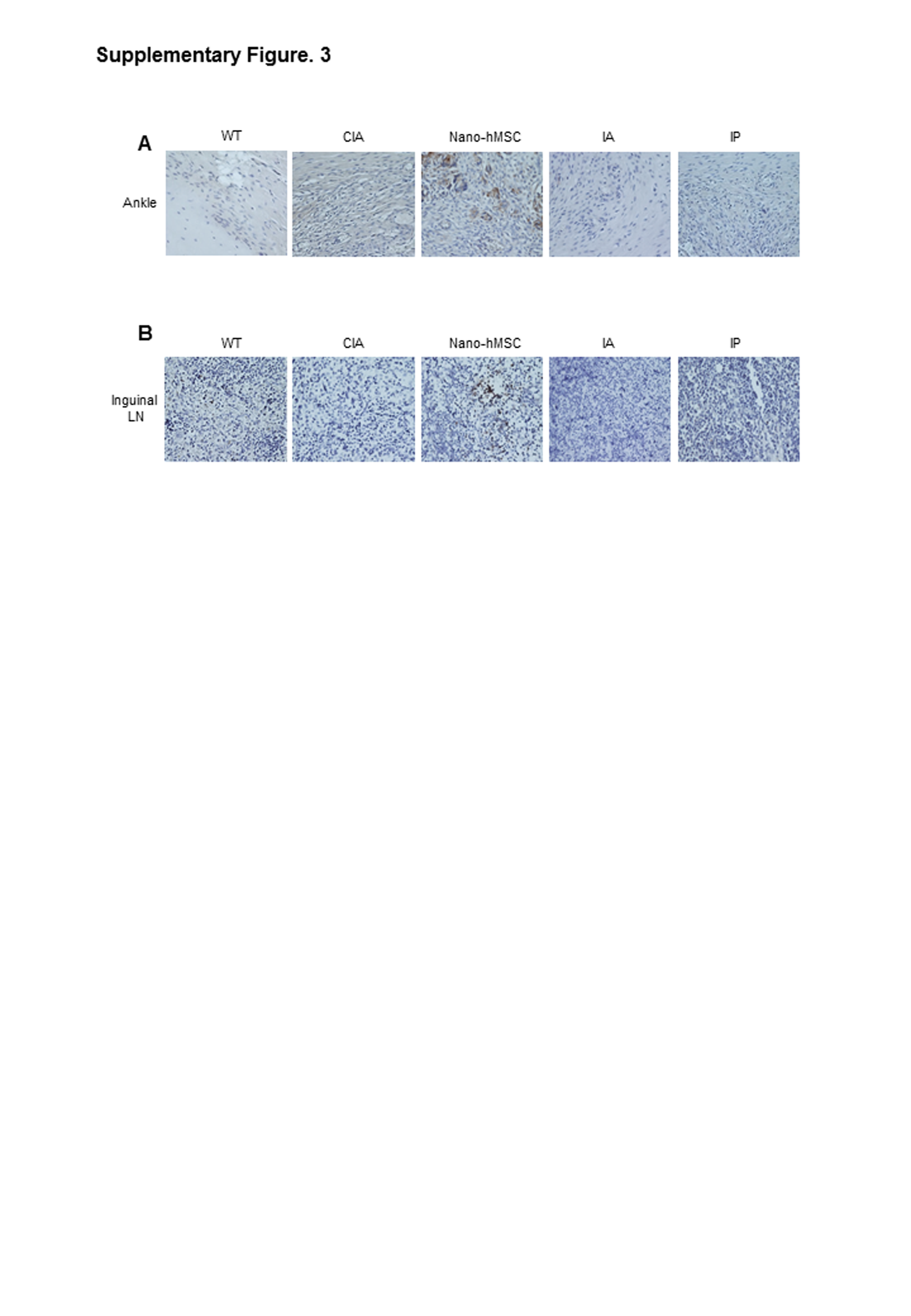

Supplement: Figure S3 — Implantation of nano-fiber induce Foxp3+ cells. CIA rats were induced and treated as indicated. Foxp3+ cells were detected by immunohistochemistry staining in (A): ankles and (B): inguinal LN at 2 weeks. Representative pictures from 3 independent experiments, original magnification×400. (TIF) [file pone.0114621.s003.tif]
